# Signaling Network Response to α-Particle–Targeted Therapy with the ^225^Ac-Labeled Minigastrin Analog ^225^Ac-PP-F11N Reveals the Radiosensitizing Potential of Histone Deacetylase Inhibitors

**DOI:** 10.2967/jnumed.122.264597

**Published:** 2023-06

**Authors:** Yun Qin, Stefan Imobersteg, Stephan Frank, Alain Blanc, Tanja Chiorazzo, Philipp Berger, Roger Schibli, Martin P. Béhé, Michal Grzmil

**Affiliations:** 1 Center for Radiopharmaceutical Sciences, Paul Scherrer Institute, Villigen, Switzerland;; 2 Department of Chemistry and Applied Biosciences, ETH Zurich, Zurich, Switzerland;; 3 Division of Neuropathology, Institute of Pathology, University of Basel, Basel, Switzerland; and; 4 Laboratory of Nanoscale Biology, Paul Scherrer Institute, Villigen, Switzerland

**Keywords:** ^225^Ac, phosphoproteomics, minigastrin, CCKBR, radioresistance

## Abstract

α-particle emitters have recently been explored as valuable therapeutic radionuclides. Yet, toxicity to healthy organs and cancer radioresistance limit the efficacy of targeted α-particle therapy (TAT). Identification of the radiation-activated mechanisms that drive cancer cell survival provides opportunities to develop new points for therapeutic interference to improve the efficacy and safety of TAT. **Methods:** Quantitative phosphoproteomics and matching proteomics followed by the bioinformatics analysis were used to identify alterations in the signaling networks in response to TAT with the ^225^Ac-labeled minigastrin analog ^225^Ac-PP-F11N (DOTA-(dGlu)_6_-Ala-Tyr-Gly-Trp-Nle-Asp-Phe) in A431 cells, which overexpress cholecystokinin B receptor (CCKBR). Western blot analysis and microscopy verified the activation of the selected signaling pathways. Small-molecule inhibitors were used to validate the potential of the radiosensitizing combinatory treatments both in vitro and in A431/CCKBR tumor–bearing nude mice. **Results:** TAT-induced alterations were involved in DNA damage response, cell cycle regulation, and signal transduction, as well as RNA transcription and processing, cell morphology, and transport. Western blot analysis and microscopy confirmed increased phosphorylations of the key proteins involved in DNA damage response and carcinogenesis, including p53, p53 binding protein 1 (p53BP1), histone deacetylases (HDACs), and H2AX. Inhibition of HDAC class II, ataxia-telangiectasia mutated (ATM), and p38 kinases by TMP269, AZD1390, and SB202190, respectively, sensitized A431/CCKBR cells to ^225^Ac-PP-F11N. As compared with the control and monotherapies, the combination of ^225^Ac-PP-F11N with the HDAC inhibitor vorinostat (suberoylanilide hydroxamic acid, SAHA) significantly reduced the viability and increased the DNA damage of A431/CCKBR cells, led to the most pronounced tumor growth inhibition, and extended the mean survival of A431/CCKBR xenografted nude mice. **Conclusion:** Our study revealed the cellular responses to TAT and demonstrated the radiosensitizing potential of HDAC inhibitors to ^225^Ac-PP-F11N in CCKBR-positive tumors. This proof-of-concept study recommends development of novel radiosensitizing strategies by targeting TAT-activated and survival-promoting signaling pathways.

## Visual Abstract

**Figure:**
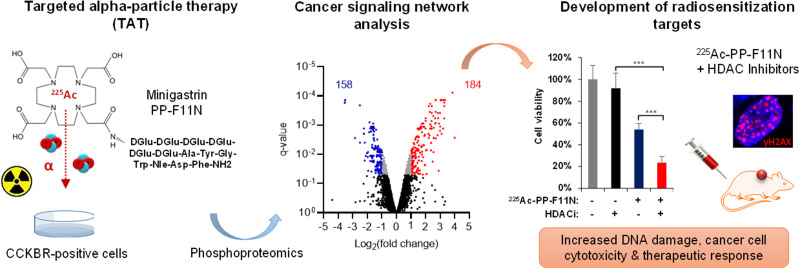

Targeted radionuclide therapy delivers cytotoxic radionuclides to cancer lesions and shows promise for the treatment of patients with unresectable metastatic cancers (1). In 2018, the Food and Drug Administration approved Lutathera (^177^Lu-labeled dotatate peptide; Advanced Accelerator Applications) as a first-in-class peptide receptor radionuclide therapy for somatostatin receptor–positive gastroenteropancreatic and neuroendocrine tumors. More recently, the ^177^Lu-labeled prostate-specific membrane antigen (PSMA) ligand ^177^Lu-PSMA-617 (Pluvicto; Advanced Accelerator Applications) has been approved for the treatment of PSMA-positive metastatic castration-resistant prostate cancer patients (2*,*^3^).

To improve therapeutic efficacy, previous studies used *α*-emitters such as ^225^Ac, with high linear energy transfer and a low tissue-penetrating range (40−100 µm) (4). Despite promising therapeutic outcomes, the effectiveness of targeted *α*-particle therapy (TAT) requires further optimization due to the impaired life quality of treated patients (5). Understanding the responses of cancer cells to TAT would allow the development of radiosensitization strategies with improved therapeutic efficacy at lower activities and reduced side effects.

We have recently developed the ^225^Ac-labeled minigastrin analog ^225^Ac-PP-F11N, which targets overexpressed cholecystokinin B receptor (CCKBR) in various human cancers including medullary thyroid, ovarian, and small-cell lung cancer, as well as gliomas (6). In a pilot and a phase I study (NCT02088645), ^177^Lu-PP-F11N demonstrated medullary thyroid cancer–specific accumulation and low retention in kidney and bone marrow, whereas the median tumor-to-stomach dose ratio of 3.34 indicated stomach as a potential dose-limiting organ (7). To understand cellular responses to ionizing irradiation caused by *α*-particle–emitting radiolabeled minigastrin, and to further develop concomitant radiosensitizing strategies, we analyzed signaling networks in response to ^225^Ac-PP-F11N in A431/CCKBR cells by quantitative phosphoproteomics and corresponding proteomics analysis.

The current study translates acquired basic radiobiology knowledge into novel treatment opportunities and provides proof of concept for the development of radiosensitizing strategies for targeted radionuclide therapies.

## MATERIALS AND METHODS

### Reagents and Radiolabeling

The selective inhibitors 7-fluoro-1-isopropyl-3-methyl-8-(6-(3-(piperidin-1-yl)propoxy)pyridin-3-yl)-1H-imidazo[4,5-c]quinolin-2(3H)-one (AZD1390) (ataxia-telangiectasia mutated [ATM]), *N*-[[tetrahydro-4-(4-phenyl-2-thiazolyl)-2H-pyran-4-yl]methyl]-3-[5-(trifluoromethyl)-1,2,4-oxadiazol-3-yl]-benzamide (TMP269) (class IIa histone deacetylase [HDAC]), 4-(4-fluorophenyl)-2-(4-hydroxyphenyl)-5-(4-pyridyl)-1H-imidazole (SB202190) (p38α and p38β2), and suberoylanilide hydroxamic acid (SAHA) (class II, III, and IV HDAC) were obtained from Lucerna-Chem. ^225^Ac (in 0.1 M HCl) was purchased from ITG GmbH, whereas the N-terminal DOTA-conjugated gastrin analog PP-F11N (DOTA-(dGlu)_6_-Ala-Tyr-Gly-Trp-Nle-Asp-Phe) was from PSL GmbH. Radiolabeling and separation of ^225^Ac-PP-F11N are described in the supplemental materials (available at http://jnm.snmjournals.org) (6).

### Cell Culture and Proliferation Assay

Human squamous carcinoma A431 cells, which overexpress CCKBR, were kindly provided by Dr. Luigi Aloj (8) and cultured under standard conditions, and the cell proliferation was analyzed using the CellTiter 96 AQueous Non-Radioactive Cell Proliferation Kit (Promega) according to the manufacturer’s instruction as described in the supplemental materials.

### Proteomics, Phosphoproteomics, and Bioinformatics

The supplemental materials describe preparation of tryptic peptides, phosphopeptide enrichment, liquid chromatography–mass spectrometry analysis, protein and phosphopeptide identification, label-free quantification, and bioinformatics (9).

### Western Blot and Immunocytochemistry

For the analysis of protein level and phosphorylation, cells were subjected to Western blot analysis and immunocytochemistry as described in the supplemental materials.

### In Vivo Therapy Study

All experiments involving mice complied with Swiss animal protection laws and were approved by the Cantonal Committee of Animal Experimentation (license 75699, 2017). Immunocompromised CD-1 female nude mice (Charles Rivers) were implanted with 5 million A431/CCKBR cells via subcutaneous injection. Seven days after inoculation, the mice carrying A431/CCKBR tumors were randomly grouped (the average tumor volume per group was 0.13 cm^3^; range, 0.11–0.14 cm^3^) and received 10 daily 50 mg/kg doses of SAHA (dissolved in dimethylsulfoxide/polyethylene glycol 400/polysorbate80/saline [10:40:5:45]) or vehicle control via intraperitoneal injection. The SAHA dose was based on the previous animal studies, which showed antitumor activity without detectable toxicity (10).

On the second day of treatment, a 30-kBq dose of ^225^Ac-PP-F11N dissolved in 100 µL of phosphate-buffered saline, or phosphate-buffered saline alone as a vehicle control, was injected intravenously. Tumor diameter, animal weight, and animal well-being were recorded at least 3 times a week, and the tumor volume was calculated as (width^2^ × length)/2. The mice were killed when the tumor reached the endpoint volume (>1.5 cm^3^). Mice with ulcerated tumors, found randomly in all groups, were killed prematurely and were excluded from the study.

For the histopathologic assessment, postmortem-dissected stomach and kidney were formalin-fixed, dehydrated, and paraffin-embedded for preparation of hematoxylin- and eosin-stained tissue sections as described previously (11). Image analysis and documentation were performed using a slide scanner (Nikon Instruments Europe).

### Statistics

Nonparametric Mann–Whitney unpaired testing and the Bliss independence model were used for in vitro treatments and calculations of combination index. In vivo, 1-way ANOVA followed by Tukey multiple-comparison testing were performed for 3 or more groups using GraphPad Prism, version 7.00, for Microsoft Windows, version 10. For survival analysis, Gehan–Breslow–Wilcoxon testing was performed. Values of *P* less than 0.05 were considered statistically significant.

## RESULTS

### Signaling Network Changes in Response to TAT with ^225^Ac-PP-F11N

**FIGURE 1.**
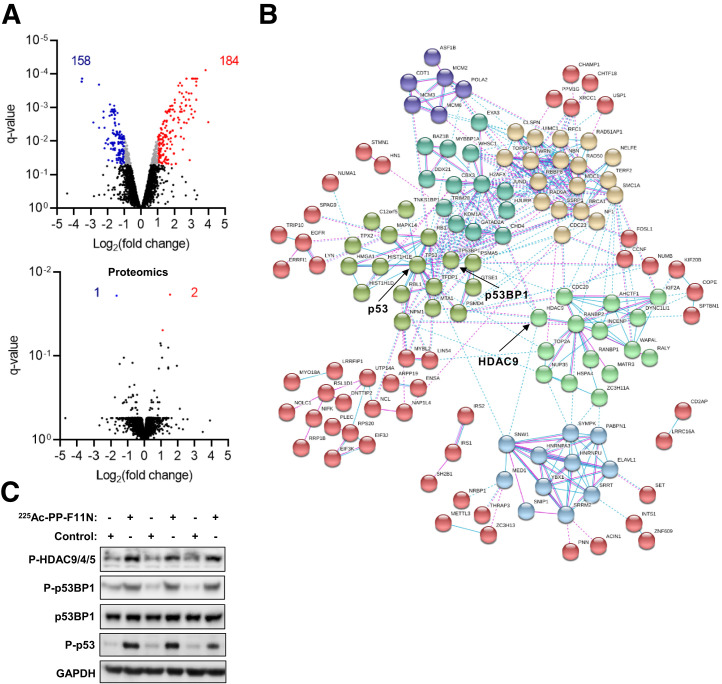
Cellular responses to TAT with ^225^Ac-PP-F11N. (A) A431/CCKBR cells were treated with ^255^Ac-PP-F11N, and the generated tryptic peptides and phosphopeptide-enriched samples were subjected to proteomics and phosphoproteomics analysis, respectively. Volcano plots display phosphopeptide (phosphoproteomics) and protein (proteomics) abundance shown as log2-transformed fold change. Red and blue dots indicate significantly altered abundance of phosphopeptides or proteins. Q-value < 0.05. (B) Interaction networks of proteins with altered phosphorylation or expression in response to ^225^Ac-PP-F11N treatment. (C) Western blot analysis for phosphorylation of HDAC9, HDAC4, and HDAC5 at S246, S259, and S220, respectively; p53BP1 at S1778; p53 at S15; and total p53BP1 and GAPDH in protein lysates isolated from ^225^Ac-PP-F11N–treated and untreated (control) cells.

We performed quantitative phosphoproteomics and proteomics analysis of the protein lysates derived from the control and ^225^Ac-PP-F11N–treated A431/CCKBR cells to identify molecular changes in response to the ^225^Ac-labeled minigastrin analog. Phosphoproteomics quantified the abundance of 8952 phosphopeptides, whereas matching proteomics quantified 4250 protein groups (Fig. 1A). The phosphoproteomics and proteomics analysis identified 342 phosphopeptides (Supplemental Tables 1 and 2) and 3 proteins (Supplemental Table 3), respectively, with significantly altered abundance in the ^225^Ac-PP-F11N–treated cells as compared with control cells. Bioinformatics analysis using the STRING platform identified the interaction networks among the proteins with altered levels of phosphorylation in the ^225^Ac-PP-F11N–treated cells (Fig. 1B). The increased phosphorylation of HDAC9, HDAC4, and HDAC5 at S246, S259, and S220, respectively; p53 binding protein 1 (p53BP1) at S1778; and p53 at S15 was validated by Western blot analysis using phosphospecific antibodies (Fig. 1C). The total protein level of p53BP1 and housekeeping protein GAPDH showed no significant difference. Further bioinformatics analysis using the DAVID web-based platform identified fold enrichment for the biologic processes, including DNA damage response (DDR), cell cycle regulation, and signal transduction pathways (Table 1), as well as RNA transcription and processing, cell morphology and adhesion, and protein modifications and transport (Supplemental Table 4).

**TABLE 1.** Significantly Enriched (*P* < 0.01) Biologic Processes and Signal Transduction Pathways in Response to ^225^Ac-PP-F11N Treatment

^225^Ac-PP-F11N	Fold enrichment	*P*
DDR, repair and nucleus structure		
DNA replication (BRCA1, POLA2, RAD50, RAD9A, RBBP8, SET, TICRR, WRN, CDT1, CHTF18, CLSPN, MCM2, MCM3AP, MCM3, MCM6, NBN, RFC1, SSRP1, TOPBP1) GOTERM_BP	9.1	3.1E−12
DNA repair (BRCA1, RAD50, RAD51AP1, RAD9A, RBBP8, TICRR, WRN, BOD1L1, CLSPN, NBN, NPM1, SMC1A, SSRP1, TOPBP1, TRIM28) GOTERM_BP	4.7	3.8E−6
Double-strand break repair via nonhomologous end joining (BRCA1, H2AFX, RAD50, WHSC1, MDC1, NBN, TP53BP1, UIMC1) GOTERM_BP	9.4	2.1E−5
Double-strand break repair via homologous recombination (BRCA1, H2AFX, RAD50, RAD51AP1, RBBP8, XRCC1, NBN, NUCKS1) GOTERM_BP	8.0	6.1E−5
Strand displacement (BRCA1, RAD50, RAD51AP1, RBBP8, WRN, NBN) GOTERM_BP	17.1	2.2E−5
DNA damage checkpoint (H2AFX, RAD9A, CLSPN, MAPK14, NBN, TP53BP1) GOTERM_BP	14.8	4.6E−5
DNA synthesis involved in DNA repair (BRCA1, RAD50, RAD51AP1, RBBP8, WRN, NBN) GOTERM_BP	12.7	9.9E−5
DNA unwinding involved in DNA replication (HMGA1, MCM2, MCM6, TOP2A) GOTERM_BP	29.6	2.7E−4
DNA double-strand break processing (BRCA1, RAD50, RBBP8, NBN) GOTERM_BP	19.7	9.7E−4
DNA duplex unwinding (RAD50, WRN, CHD4, MCM3, NBN) GOTERM_BP	8.4	2.8E−3
Nucleosome assembly (H2AFX, SET, ASF1B, HIST1H1D, HIST1H1E, MCM2, NPM1, NAP1L4) GOTERM_BP	5.0	1.1E−3
Telomere maintenance via telomerase (RAD50, RFC1, TNKS1BP1, TERF2) GOTERM_BP	16.4	1.7E−3
Covalent chromatin modification (RB1, RBL1, ASF1B, CBX3, C17orf49, TRIM28, ZMYND11) GOTERM_BP	4.6	4.3E−3
Telomere maintenance via recombination (POLA2, RAD50, WRN, RFC1) GOTERM_BP	9.2	9.0E−3
Cell cycle regulation		
Cell division (CD2AP, RBBP8, RB1, TPX2, TRIOBP, WAPL, ARPP19, CDC20, CDC23, CDCA2, CCNF, DYNC1LI1, ENSA, HELLS, KIF20B, KIF2A, MAP4, MISP, NUMA1, PSRC1, PKN2, SMC1A, ZC3HC1) GOTERM_BP	4.9	2.2E−9
Mitotic nuclear division (CD2AP, RBBP8, TPX2, TRIOBP, ARPP19, CDC20, CDC23, CDCA2, CCNF, DYNC1LI1, ENSA, HELLS, INCENP, KIF20B) GOTERM_BP	5.4	4.5E−8
Meiotic cell cycle (H2AFX, RBBP8, RBM7, NBN, NUMA1, ZNF318) GOTERM_BP	13.1	8.6E−5
Cell cycle (BRCA1, HJURP, RBL1, CDC20, CHTF18, LIN54, MCM2, NOLC1, PKN2, TERF2, TP53, ZMYND11) GOTERM_BP	4.1	1.8E−4
G1/S transition of mitotic cell cycle (POLA2, RANBP1, RBBPB8, RB1, CDT1, MCM2, MCM3, MCM6) GOTERM_BP	5.8	4.6E−4
Regulation of cell cycle (JUND, MYBL2, RB1, RBL1, CCNF, FIGNL1, LIN54, MED1) GOTERM_BP	4.8	1.4E−3
G2 DNA damage checkpoint (BRCA1, RBBP8, CLSPN, UIMC1) GOTERM_BP	14.8	2.3E−3
Cell cycle checkpoint (RBBP8, RB1, TICRR) GOTERM_BP	24.7	6.1E−3
Mitotic cell cycle checkpoint (RB1, TTK, NBN, SMC1A) GOTERM_BP	9.2	9.0E−3
Mitotic spindle organization (TTK, KIF2A, MAP4, STMN1, SMC1A) GOTERM_BP	12.3	6.7E−4
Chromosome segregation (BRCA1, HJURP, CDCA2, INCENP, PPP1R7, TOP2A) GOTERM_BP	6.5	2.2E−3
Sister chromatid cohesion (AHCTF1, RANBP2, WAPL, CDC20, INCENP, KIF2A, SMC1A) GOTERM_BP	5.0	2.7E−3
Signal transduction and cellular response		
Regulation of signal transduction by p53 class mediator (BRCA1, RAD50, RAD9A, RBBP8, TPX2, WRN, CHD4, MAPK14, NBN, SSRP1, TOPBP1, TP53) GOTERM_BP	7.2	9.2E−7
Cellular response to DNA damage stimulus (BRCA1, H2AFX, LYN, RAD50, RAD9A, TIGAR, WRN, BOD1L1, BAZ1B, TOP2A, TOPBP1, TP53BP1, TP53) GOTERM_BP	4.6	2.6E−5
Response to ionizing radiation (BRCA1, EYA3, H2AFX, TICRR, MTA1, TOPBP1, UIMC1) GOTERM_BP	10.6	4.8E−5
Cellular response to ionizing radiation (RAD51AP1, RAD9A, FIGNL1, MAPK14, TP53) GOTERM_BP	11.9	7.6E−4
Cellular response to epidermal growth factor stimulus (ERRFI1, ZFP36L2, ZFP36, EGFR, MED1) GOTERM_BP	11.2	9.6E−4
Cellular response to dexamethasone stimulus (ERRFI1, CBX3, EGFR, HNRNPU) GOTERM_BP	10.2	6.8E−3
DDR, signal transduction by p53 class mediator resulting in cell cycle arrest (GTSE1, NPM1, TNKS1BP1, TFDP1, TP53) GOTERM_BP	6.0	9.7E−3
ATM signaling pathway (BRCA1, RAD50, RBBP8, NBN, TP53) BIOCARTA	8.6	2.0E−3
Role of BRCA1, BRCA2, and ATM- and Rad3-related in cancer susceptibility (BRCA1, RAD50, RAD9A, NBN, TP53) BIOCARTA	8.2	2.4E−3

### Targeting TAT-Induced Pathways Sensitizes Cancer Cells to ^225^Ac-PP-F11N

**FIGURE 2.**
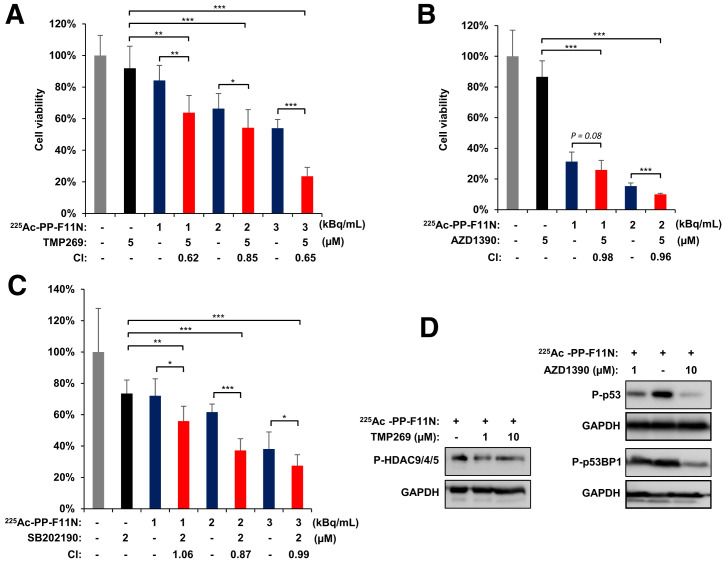
Treatment with HDAC, p38, and ATM inhibitors sensitizes A431/CCKBR cells to ^225^Ac-PP-F11N. Cell viability 48 h after treatment with ^225^Ac-PP-F11N alone or in combination with HDAC inhibitor TMP269 (A), ATMi AZD1390 (B), and p38i SB202190 (C). Bars represent mean ± SD. Corresponding combination index values between 0.9 and 1.1 indicate additive effects, and below 0.9 indicates synergism. (D) Western blot analysis for phosphorylation of HDAC9, HDAC4, and HDAC5 at S246, S259, and S220, respectively; p53 at S15; and p53BP1 at S1778 in protein lysates isolated from treated and control cells. Western blots were reprobed with antibody against GAPDH. *P < 0.05. **P < 0.01. ***P < 0.001.

We investigated inhibition of ^225^Ac-PP-F11N–activated signaling pathways to explore novel strategies for radiosensitization of TAT, previously reported to be associated with radioresistance or survival. We selected 3 druggable pathways—HDAC class II, ATM, and p38—which can be targeted by the commercially available selective small-molecule inhibitors TMP269, AZD1390, and SB202190, respectively. For the combinatory treatments, the optimal inhibitor concentration was determined in A431/CCKBR cells, whereby 5 µM of TMP269, 5 µM of AZD1390, and 2 µM of SB202190 reduced cell proliferation to 69%–89% of control (Supplemental Fig. 1). Concomitant treatment of A431/CCKBR cells with different doses of ^225^Ac-PP-F11N and TMP269, AZD1390, or SB202190 reduced cell proliferation from 63% to 23%, from 14% to 8%, or from 32% to 23% of control, respectively, and was significantly lower (*P* < 0.05) than with the monotherapy with ^225^Ac-PP-F11N or inhibitor alone (Figs. 2A–2C). The combination of ^225^Ac-PP-F11N with TMP269 showed a synergistic effect (CI, 0.62–0.85), whereas moderate synergistic and additive effects were obtained for SB202190 and AZD1390, with a combination index of 0.81–0.99 and 0.96–0.98, respectively. The inhibitions of HDAC9, HDAC4, and HDAC5 phosphorylation at S246, S259, S220, respectively; of p53 at S15; and of p53BP1 at S1778 in response to TMP269 and AZD1390 treatment were determined by Western blot analysis in ^225^Ac-PP-F11N–treated cells (Fig. 2D).

### HDAC Inhibitor SAHA Improves Therapeutic Efficacy of ^225^Ac-PP-F11N

**FIGURE 3.**
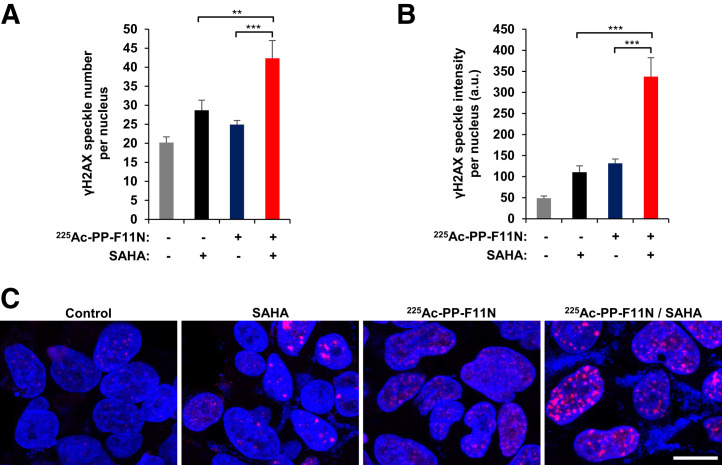
HDAC inhibition by SAHA increased level of γH2AX in ^225^Ac-PP-F11N–treated cells. A431/CCKBR cells were treated with 3 kBq/mL dose of ^225^Ac-PP-F11N or 2 μM SAHA alone or in combination for 24 h. (A and B) Bars represent mean ± SEM of numbers and intensities of γH2AX-positive speckles per nucleus. (C) Typical images of treated and control cells. Red = γH2AX; blue = Hoechst 33258; scale bar = 20 μm; a.u. = arbitrary units. **P < 0.01. ***P < 0.001.

**FIGURE 4.**
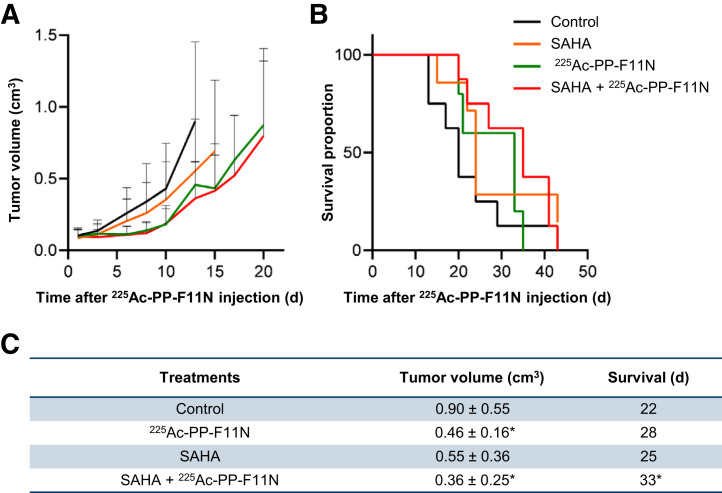
Tumor growth inhibition and prolonged survival in SAHA and ^225^Ac-PP-F11N–treated mice. (A) Tumor growth curves in A431/CCKBR xenografted mice after administration of ^225^Ac-PP-F11N or phosphate-buffered saline (control) alone or in combination with SAHA. Values represent mean ± SD. (B) Survival proportion presented as Kaplan–Meier curves of control and different treatment groups. (C) Mean tumor volume ± SD on day 13 and survival in control and different treatment groups. *P < 0.05.

**FIGURE 5.**
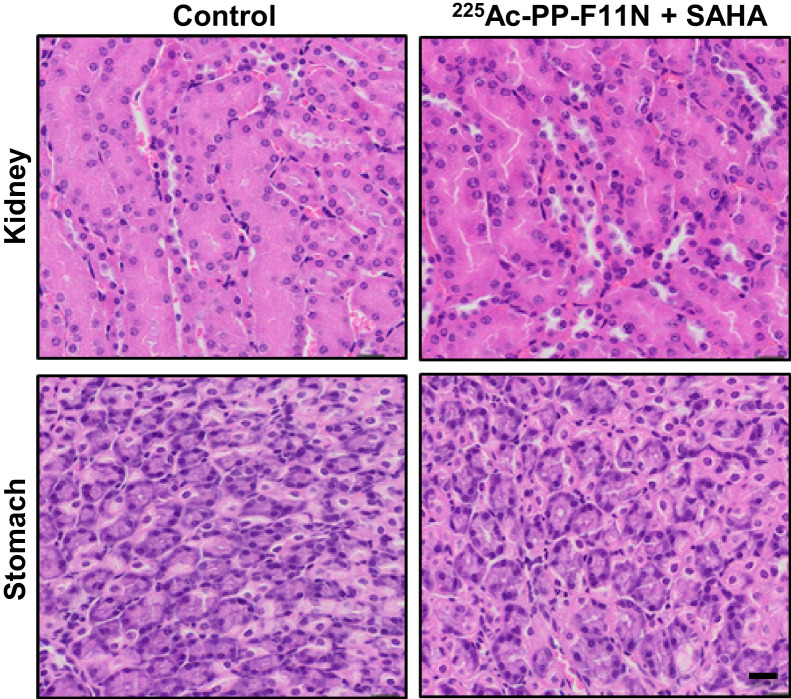
Histology of kidney and stomach. Representative images of tissue sections stained with hematoxylin and eosin of kidney and stomach isolated from control and ^225^Ac-PP-F11N and SAHA-treated mice 35–43 d after injection of activity. Scale bar = 20 μm.

In a search for novel radiosensitizing approaches for ^225^Ac-PP-F11N, we selected the Food and Drug Administration–approved HDAC inhibitor SAHA, which inhibited cell proliferation to 74% of control at 2 µM (Supplemental Fig. 1). We analyzed the DNA double-strand break marker *γ*H2AX (H2AX phosphorylation at S139) to investigate effects on the DNA damage, which expression correlated with the response to targeted radionuclide therapy (12). The combination of ^225^Ac-PP-F11N and SAHA showed a significantly increased speckle number and intensity of *γ*H2AX in the nucleus (Figs. 3A–3C) and reduced A431/CCKBR cell viability (Supplemental Fig. 2) as compared with the monotherapies and control. A431/CCKBR tumor–bearing nude mice were analyzed after administration of a daily 50 mg/kg dose of SAHA for 10 d, alone or in combination with a single 30-kBq dose of ^225^Ac-PP-F11N. All treatments delayed tumor growth (Fig. 4A). The first mouse reached the endpoint in the control group on day 13 after ^225^Ac-PP-F11N application, and the average tumor volumes in the ^225^Ac-PP-F11N and combinatorial treatment groups were significantly reduced to 0.46 cm^3^ (*P* = 0.04) and 0.36 cm^3^ (*P* = 0.02), respectively, as compared with control (0.90 cm^3^). Treatment with SAHA reduced the average tumor volume to 0.55 cm^3^ (*P* = 0.12). The mean survival of mice treated with SAHA and ^225^Ac-PP-F11N was significantly extended (33 d, *P* = 0.04) as compared with the control (22 d) (Figs. 4B and 4C). In contrast, monotherapies with ^225^Ac-PP-F11N or SAHA extended mean survival to 28 and 25 d, respectively, but these results did not reach statistical significance. To investigate potential toxicity to healthy organs, we analyzed the kidney, involved in circulating radiopeptide excretion, and the stomach, accumulating ^225^Ac-PP-F11N because of endogenous CCKBR expression (6). Histopathologic assessment of kidney and stomach tissue sections from mice treated with SAHA and ^255^Ac-PP-F11N did not show any differences from controls (3 mice per group) (Fig. 5). Furthermore, during therapy, no body weight loss was observed in any treatment group (Supplemental Fig. 3).

## DISCUSSION

Despite new advances in TAT, cancer radioresistance remains a challenge that worsens therapeutic outcomes in the clinic (13*,*^14^). To identify radiosensitizing molecular targets and to develop combinatory treatments, we characterized changes in the cancer signaling network in response to peptide receptor radionuclide therapy with the ^225^Ac-labeled minigastrin analog ^225^Ac-PP-F11N. Understanding cancer cell responses can result in the coherent design of radiosensitization strategies to improve the therapeutic window, reduce applied activity, and, thus, minimize adverse effects. This rational approach can be also applied to other radioconjugates to develop safer and more efficacious cancer treatments.

Our phosphoproteomic analysis identified phosphorylation changes in proteins involved in DDR, repair, and nucleus structure, as well as in cell cycle regulation, RNA processing, and signal transduction. Consistently, ionizing radiation leads to the formation of DNA damage foci and activation of DDR pathways via activation of ATM/checkpoint kinase 2 and ATM- and Rad3-related/checkpoint kinase 1, which regulate proteins involved in DNA repair, cell cycle progression, and chromatin regulation and gene expression (1). Although mass spectrometry–based quantitative characterization of proteome and posttranslational modifications was previously used in the prediction of drug responses (15), the identification of cancer biomarkers, and the identification of sensitization targets for external-beam radiation therapy (16*,*^17^), little is known about cancer responses to targeted radionuclide therapy.

Recently, mass spectrometry–based phosphoproteomics analyzed altered signaling networks in response to targeted radioligand therapy with ^177^Lu- and ^255^Ac-labeled PSMA in a prostate cancer mouse model (18). Similarly, the study identified alterations in DNA damage and replication stress response as well as in p53 pathways and suggests that the identified pathways may mediate radioresistance, yet the validation and development of radiosensitizing strategies await further investigation. Despite similarities in the response to targeted radionuclide therapy, the genetic heterogeneity of various cancers influences activation of the signaling pathways, and thus, effective radiosensitization might require development of cancer-type–specific strategies. Among identified alterations, our validation study confirmed increased phosphorylation of HDAC9, HDAC4, and HDAC5 at S246, S259, and S220, respectively, as well as p53BP1 at S1778 and p53 at S15 in response to ^225^Ac-PP-F11N. HDACs play a role in the chromatin remodeling and regulation of posttranscriptional gene expression, which are essential processes in DDR (19).

The phosphorylation of HDAC regulates nucleocytoplasmic shuttling, complex formation, and catalytic activity (20*,*^21^). Notably, Biade et al. reported chromatin conformation changes after cotreatment with the HDAC inhibitor trichostatin A and external-beam radiation therapy, which led to enhanced radiation sensitivity in intrinsically radioresistant colon carcinoma cells (22). Consistently in our study, the combination of HDAC inhibitor with TAT resulted in a synergistic effect on cell viability inhibition, which could be explained by the enhanced number of DNA double-strand breaks. In addition, the chromatin modulators, including demethylating agents and HDAC inhibitors, were reported to upregulate SSTR2 expression and thus increased tumor uptake of the radiolabeled octreotide in neuroendocrine and prostate cancer cells (23). An assessment of whether these findings also apply to other targeted receptors, including CCKBR, requires further investigation.

The ATM-phosphorylated p53BP1 acts as a sensor protein of DNA damage and is involved in recruiting repair proteins to the damaged chromatin (24). Interaction of p53BP1 with the telomere-associated protein RIF1 potentiated cell survival after multifractionated radiotherapy, and this survival benefit can be revoked by p53BP1 inhibition (25). Furthermore, the elevated phosphorylation level of the tumor suppressor p53 on serine-15 after ionizing radiation has been reported to mediate cell growth arrest, which provides time to facilitate DNA repair (26*,*^27^). Our phosphoproteomics identified increased phosphorylation of MAPK14 (p38 isoform α), which regulates various biologic responses including proliferation, differentiation, migration, and inflammation, as well as stress responses and survival (28–30). Notably, Rac1-mediated p38 activation in response to γ-rays supported cervical carcinoma cell survival, and the inhibition of Rac1 activity abrogated the radioresistance conferred by Rac1/p38 activation and significantly enhanced apoptosis (31). Thus, the important roles of the HDACs, ATM/p53, and p38 pathways in DDR and survival, previously reported and identified by our study, increased phosphorylations in response to TAT, pointing them to potential radiosensitizing targets.

Indeed, in the present study, inhibition of the HDAC class II, ATM, and p38 pathways by small-molecule inhibitors significantly enhanced the cytotoxic effect of ^225^Ac-PP-F11N in CCKBR-positive cells. As expected, interference with DDR pathways by ATMi AZD1390 sensitized cancer cells to ionizing radiation, whereas p38i showed a weaker radiosensitizing effect than HDAC inhibitor, which showed the synergistic effect with ^225^Ac-PP-F11N. Thus, in a search for the most efficient radiosensitizing strategy for clinical applications, we selected for in vivo validation the HDAC inhibitor SAHA (vorinostat), which is approved by the Food and Drug Administration for the treatment of cutaneous T-cell lymphoma patients (32). In addition, SAHA showed better anticancer activity than the other HDAC inhibitor, TMP269, and significantly enhanced the DNA damage and cytotoxicity of ^225^Ac-PP-F11N in our in vitro assays. As compared with the monotreatment and control groups, SAHA in combination with ^225^Ac-PP-F11N produced the most effective therapeutic response in vivo.

This first proof-of-concept study confirms the radiosensitizing potential of HDAC inhibitors, yet to maximize therapeutic response this study requires further optimization. In agreement with our results, the radiosensitization effects of the HDAC inhibitor were previously reported in various cancer models (19), and more recently, cotreatment with vorinostat improved the response to the radiolabeled peptide ligand ^212^Pb-DOTA-MC1L in mice bearing human melanoma xenografts (33). Furthermore, in CD1 nude mice human xenografted with RT112 bladder cancer, radiotherapy in combination with the HDAC inhibitor panobinostat delayed cancer growth without significantly increasing acute and short-term normal-tissue radiation toxicity (34). Similarly, in our study, neither acute radiation toxicity to the kidney or stomach nor significant body weight losses were identified in mice that received combinatory treatment, indicating that the applied doses of ^225^Ac-PP-F11N and SAHA were relatively safe and well tolerated. Moreover, the combination of vorinostat with external radiotherapy has recently entered clinical trials with non–small cell lung cancer (NCT00821951) and glioblastoma (NCT03426891) patients to assess safety, tolerability, and efficacy, thus suggesting that HDAC inhibitor treatment is a clinically feasible radiosensitizing strategy for TAT in cancer patients.

## CONCLUSION

Our phosphoproteomic analysis followed by a validation study revealed alterations in the signaling networks and identified radiosensitizing molecular targets, including HDAC, ATM, and p38, in response to TAT with a ^225^Ac-labeled minigastrin analog in CCKBR-positive cancer cells. In this study, the explored radiobiology was used to verify new radiosensitizing strategies based on the targeting radiation-activated and survival-supporting pathways. The combination of ^225^Ac-PP-F11N with the HDAC inhibitor vorinostat enhanced DNA damage and cancer cell cytotoxicity and improved therapeutic efficacy in A431/CCKBR tumor–bearing nude mice. Our proof-of-concept study indicates that HDAC inhibitor treatment is an effective radiosensitization strategy for ^225^Ac-PP-F11N and further recommends phosphoproteomics for the identification of novel radiosensitizing targets.

## DISCLOSURE

This research was supported by funds from the Swiss Cancer League (KFS-3960-08-2016-R) to Michal Grzmil, Martin Béhé, and Roger Schibli. Martin Béhé and Roger Schibli are listed as inventors on patent WO2015/067473: “Mini-Gastrin Analog, in Particular for Use in CCK2 Receptor Positive Tumor, Diagnosis or Treatment.” No other potential conflict of interest relevant to this article was reported.

## Notes

KEY POINTS **QUESTION:** How do cancer cells respond to TAT, and which survival-supporting pathways are potential molecular targets for the rational development of radiosensitizing strategies? **PERTINENT FINDINGS:** TAT with ^255^Ac-PP-F11N induced DDR, cell cycle regulation, RNA transcription and processing, as well as signal transduction pathways in treated cancer cells. Targeting of identified HDAC, ATM, and p38 pathways shows radiosensitizing potential in cancer cells, and the clinically approved HDAC inhibitor vorinostat (SAHA) significantly improves the efficacy of TAT in vivo. **IMPLICATIONS FOR PATIENT CARE:** Patients with CCKBR-positive tumors might benefit from combinatory treatment with HDAC inhibitor and a radioactive minigastrin analog because of the enhanced radiosensitivity and anticancer activity of the HDAC inhibitor.
